# Evaluation of the tuberculosis culture color plate test for rapid detection of drug susceptible and drug-resistant *Mycobacterium tuberculosis* in a resource-limited setting, Addis Ababa, Ethiopia

**DOI:** 10.1371/journal.pone.0215679

**Published:** 2019-05-28

**Authors:** Biruk Mekonnen, Adane Mihret, Muluwork Getahun, Tsegaye Hailu, Sabeen Sidiki, Holden V. Kelley, Julia M. Scordo, W. Garrett Hunt, Xueliang Pan, Joan-Miquel Balada-Llasat, Wondwossen Gebreyes, Carlton A. Evans, Abraham Aseffa, Jordi B. Torrelles, Shu-Hua Wang, Tamrat Abebe

**Affiliations:** 1 Department of Microbiology, Immunology and Parasitology, College of Health Sciences, Addis Ababa University, Addis Ababa, Ethiopia; 2 Armauer Hansen Research Institute (AHRI), Addis Ababa, Ethiopia; 3 Ethiopia Public Health Institute (EPHI), Addis Ababa, Ethiopia; 4 Department of Microbial Infection and Immunity, College of Medicine, The Ohio State University, Columbus, Ohio, United States of America; 5 Nationwide Children’s Hospital, The Ohio State University, Columbus, Ohio, United States of America; 6 Center for Biostatistics, College of Medicine, The Ohio State University, Columbus, Ohio, United States of America; 7 Department of Pathology, College of Medicine, The Ohio State University, Columbus, Ohio, United States of America; 8 Global One Health Initiative (GOHi), The Ohio State University, Columbus, Ohio, United States of America; 9 Infectious Diseases & Immunity, Imperial College London, and Wellcome Trust Imperial College Centre for Global Health Research, London, United Kingdom; 10 Innovation for Health and Development (IFHAD), Laboratory of Research and Development, Universidad PeruanaCayetano Heredia University, Lima, Peru; 11 InnovaciónPor la Salud Y Desarrollo (IPSYD), Asociación Benéfica PRISMA, Lima, Perú; 12 Division of Infectious Diseases, Department of Internal Medicine, College of Medicine, The Ohio State University, Columbus, Ohio, United States of America; Jamia Hamdard, INDIA

## Abstract

Timely diagnosis of tuberculosis (TB) is limited in Ethiopia. We evaluated the performance of a low technology, thin layer agar, *Mycobacterium tuberculosis* (*M*.*tb*) culture color plate (TB-CX) test with concurrent drug susceptibility testing (DST) to isoniazid (INH), rifampin (RIF), and pyrazinamide (PZA) directly from sputum specimens. Patients undergoing examination for TB and multidrug-resistant (MDR)-TB were enrolled in Addis Ababa, Ethiopia from March 2016 to February 2017. All subjects received a GeneXpert MTB/RIF PCR test. TB-CX test results were compared to reference Löwenstein–Jensen (LJ) culture for *M*.*tb* detection and DST for susceptibility to INH and RIF. Kappa statistic was applied to test agreement between results for TB-CX test and the reference methods, a cut-off Kappa value of 0.75 was considered as high level of agreements. A total of 137 participants were analyzed: 88 (64%) were new TB cases, 49 (36%) were re-treatment cases. The TB-CX test detected *M*.*tb* and DST in an average of 13 days compared to 50 days for the conventional DST result. The sensitivity and specificity of the TB-CX test for detecting *M*.*tb* were 94% and 98%, respectively (concordance, 96%; kappa 0.91). The sensitivity of the TB-CX test to detect drug resistance to INH, RIF, and MDR-TB was 91%, 100%, and 90% respectively. The specificity of the TB-CX test for detecting INH, RIF, and MDR-TB was 94%, 40%, and 94% respectively. Overall agreement between TB-CX test and LJ DST for detection of MDR-TB was 93%. The TB-CX test showed strong agreement with the GeneXpert test for detecting *M*.*tb* (89%, kappa 0.76) but low agreement for the detection of RIF resistance (57%, kappa 0.28). The TB-CX test was found to be a good alternative method for screening of TB and selective drug resistant-TB in a timely and cost-efficient manner.

## Introduction

Tuberculosis (TB) is the single highest infection-associated cause of mortality in the world, surpassing either HIV/AIDS or malaria [1]. In 2017, the World Health Organization (WHO) estimated that approximately 4,000 people die of TB every day. Although TB is curable, the emergence of drug-resistant-TB is a recognized challenge for global TB prevention [1, 2, 3]. The WHO estimates approximately half a million new MDR-TB cases annually, defined as exhibiting resistance to both isoniazid (INH) and rifampin (RIF) [1].

Optimized TB treatment is based on rapid detection of *Mycobacterium tuberculosis* (*M*.*tb*) and rapid reporting of drug susceptibility test (DST) results [4, 5]. Despite improved TB diagnosis and treatment, estimated to have saved 49 million lives and to have significantly reduced TB incidence rates between the years 2000 and 2015, critical diagnostic and treatment gaps persist, especially for low and middle income countries (LMIC) [1,3,6]. In 2017, about 3.6 million new TB cases were missed (gap between estimated and notified cases) and only 64% of TB cases and 25% of MDR-TB cases were confirmed with laboratory tests [1]. These gaps are largely associated with ineffective diagnostic tools and inadequate health care infrastructure [1, 3, 6].

Timely and affordable methods to detect drug-susceptible and drug resistant-TB are not available in many resource-limited settings. The acid-fast stain fails to detect more than 30% of all incident *M*.*tb* cases and is unable to detect drug resistance [7, 8]. The GeneXpertMTB/RIF test (Cepheid, Sunnyvale, CA, USA) represents a major advance in speeding up the diagnosis of TB and RIF resistance within two hours. The detection of RIF drug resistance on the GeneXpert system has been used as a surrogate marker for MDR-TB, but the test is expensive and requires laboratory infrastructure that may not be sustainable in many areas [8,9]. Solid culture-based DST can provide definitive results but it is slow and laborious, while commercial liquid culture-based DST methods can yield rapid, reliable results within 12 days after *M*.*tb* isolation, but these platforms also require expensive laboratory infrastructure, limiting wide implementation in resource-constrained settings [6, 8, 10].

Consequently, there is an urgent global need, particularly in resource-limited settings, for a simple, rapid and affordable method to diagnose drug-resistant TB. The MDR/XDR-TB Color Test (TB-CX) test is based on thin-layer agar (TLA) technology with both culture and direct DST methods incorporated into a single four quadrant agar plate. Despite limited data, the TB-CX test has demonstrated good performance for rapid detection of drug-susceptible and drug-resistant TB [5, 11–14].

The objectives of this study were to: i) evaluate the accuracy of the TB-CX test for detection of *M*.*tb* directly from sputum specimens in comparison with conventional culture using Löwenstein- Jensen (LJ) media and the molecular GeneXpert test, ii) concurrently evaluate the detection of drug resistance to INH, RIF, and PZA against a gold standard of conventional phenotypic DST using the LJ-indirect proportional method and Mycobacterial growth indicator tube (MGIT), and iii) determine the turnaround time (TAT) for concurrent *M*.*tb* DST results by the TB-CX test compared to conventional phenotypic DST methods.

## Material and methods

### Ethical statement

The study was reviewed and approved by the ethical and research review committee of Department of Microbiology, Immunology, and Parasitology, College of Health Science, School of Medicine, Addis Ababa University, Addis Ababa, Ethiopia (DRERC/003/08, February 1, 2016). Permission was also obtained from the study site hospitals. Written Informed consent was obtained from all volunteer participants and/or families and/or guardian prior to sample collection. Confidentiality for all collected data was preserved using secret codes for each participant.

### Study settings and study period

The study was conducted at the All Africa Leprosy, Tuberculosis and Rehabilitation Training Center (ALERT) and St. Peter Hospitals, Addis Ababa, Ethiopia, from March 2016 to February 2017. Both are public hospitals and provide referral services for the diagnosis and treatment of TB and MDR-TB for patients from different regional states of the country.

### Study design, participants, and sample size determination

A cross-sectional hospital-based diagnostic test validation study was conducted. Patients were eligible for inclusion if they were suspected to have TB and/or MDR-TB (new or previously treated) according to WHO definition (i.e., previously treated >1 month and/or contacts of MDR-TB patients) [15] and if they were able to submit paired morning expectorated sputum samples. Patients were excluded if they could not produce enough sputum sample (<3ml per tube) or had received anti-TB drugs therapy for greater than two weeks. Sample size was estimated based on Buderer method [16], assuming a 95% anticipated sensitivity and specificity with absolute precision of less than 5% at 95% confidence interval (CI), with prevalence of 46.3% in a study population [17] and 10% contingency. Accordingly, a total of 147 patients were enrolled in the study. In this study, three laboratory tests were included namely (1) GeneXpert MTB/RIF test (molecular test) (2) TB-CX Test (new test) and (3) LJ culture-based DST (gold standard tests).

#### GeneXpert/MTB/RIF test

One of the paired sputum samples submitted by the study participants was directly tested by the GeneXpert test at ALERT and St. Peter’s Hospital TB laboratories following the procedure according to the national GeneXpert test manual [18,19].

#### Sputa processing and inoculum preparation for culture

The other paired sputum sample was refrigerated (4–8°C) and then transported within 24 to 48hrs on ice to the Armauer Hansen Research Institute (AHRI) TB laboratory where the TB-CX test and the gold standard tests (LJ culture and LJ DST) were performed as previously described [12–14, 20, 21]. Briefly, sputa used for the TB-CX test were decontaminated using the TB-CX test disinfectant. This disinfectant is a combination of four different chemicals namely trisodium phosphate (200g), ammonium sulphate (5g), magnesium sulphate (0.5g), and ferric ammonium citrate (0.25g), and all dissolved in 1,000ml of sterile distilled water. The disinfectant was added using a 2:1 ratio of the sputum sample, and the sputum-disinfectant mixture was kept for 1.5 to 1.75 hrs. The sputum-disinfectant solution was used as the inoculums for the culture. Whereas the sputa for LJ culture were decontaminated using the modified Petroff method (1:1 ratio), centrifuged at 3,000 rpm for 15 minutes. The re-suspended sediment was inoculated on LJ medium. All *M*.*tb* isolates grown on the LJ culture were further tested for INH, RIF, and PZA DST as described [20, 21].

#### TB-CX test: Inoculation, growth and concurrent DST detection

**Fig 1:**
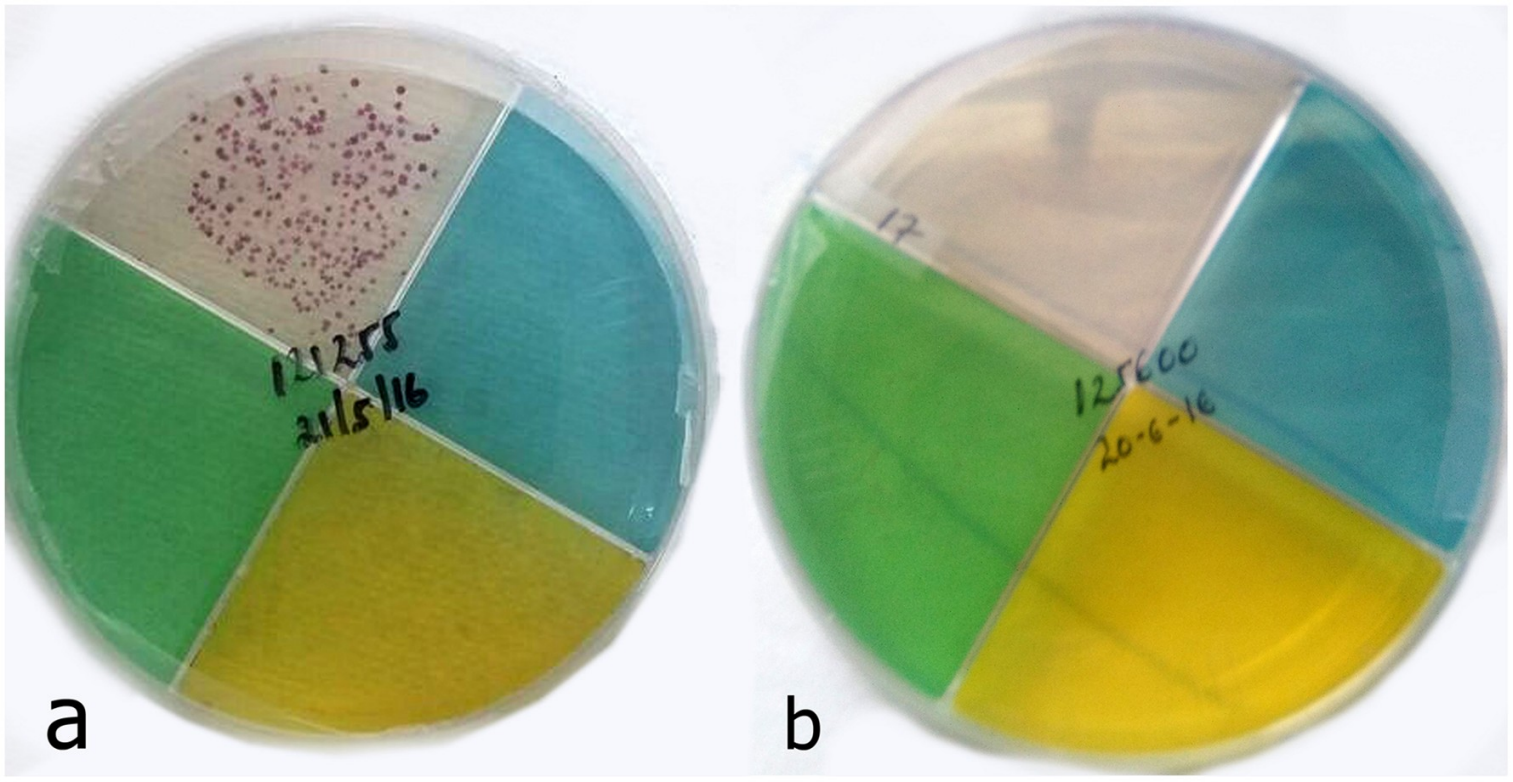
*Mycobacterium tuberculosis* (*M*.*tb*) growth detection using the TB-CX test. Positive for *M*.*tb* growth (red micro-colonies) on clear quadrant (a) and Negative for *M*.*tb* growth on clear quadrant (b).

This particular TB-CX test is based on the use of 4 distinct quadrants, with one quadrant for detection of growth (clear) and three other quadrants for DST, one quadrant each for 0.2 μg/ml INH (green), 1.0 μg/ml RIF (yellow), and 100 μg/ml PZA (blue). The TB-CX test agar plates were prepared and quality-control tested at The Ohio State University, as described previously [12], shipped to Ethiopia, and stored at 4°C up to four months until used. Each TB-CX test was inoculated using two drops (100μl) per quadrant of the disinfected sputum as described [12 –14]. The TB-CX test plate was double-sealed using Parafilm, put into a Zip-lock bag, and incubated at 37°C. Each TB-CX test was read visually three times a week until at least 50 *M*.*tb* colonies appeared in the drug-free quadrant, for a maximum of a 6 week period. *M*.*tb* growth was defined as the macroscopic appearance of red micro-colonies on the drug-free quadrant (Fig 1a). Specific identification of *M*.*tb* was based on the visualization of cording under light microscopy examination (40X) of the doubly-sealed plate [12–14, 22]. DST results in the INH, RIF and PZA quadrants were read at the same time that the growth was visualized on the drug-free quadrant. A strain was considered resistant to a particular drug when the number of colonies that appeared on the drug-containing medium was >1% of the colonies that appeared concurrently on the drug-free medium.

#### Growth detection using LJ culture

The LJ culture media were prepared as previously described [20, 21]. If no growth was detected at 8 weeks, it was recorded as negative. Contaminated samples were decontaminated and re-inoculated onto LJ culture slants. *M*.*tb* growth was confirmed by positive acid-fast bacilli (AFB) smear growth rate and colony morphology. All positive cultures were stored at -80°C for DST.

#### DST by Indirect Proportion Method (IPM) and Mycobacterial growth indicator tube (MGIT)

The conventional DST for INH and RIF was performed using IPM per established procedures on LJ medium with critical concentrations of 0.2μg/mL for INH and 40μg/mL for RIF [20, 21]. DST for PZA was performed using an automated liquid culture (Bactec MGIT 960) system [20, 21, 23] with drug concentration of 100μl/ml on 38 randomly selected *M*.*tb* isolates: 19 PZA positive with TB-CX test isolates and 19 PZA negative with TB-CX test isolate.

#### Time to result for growth detection and DST

Time to report was calculated in terms of days from the date of inoculation to the date of interpretable positive results. The number of days to positive-result availability for each TB-CX test and LJ culture and LJ DST test for INH and RIF was designated as turnaround time (TAT).

### Statistical analysis

All data were entered into and managed with SPSS version 21.0 (SPSS, Chicago, IL, USA). Descriptive statistics were analyzed. Two-by-2 contingency tables were used for calculation of diagnostic parameters, such as sensitivity, specificity, positive predictive value (PPV), negative predictive value (NPV), and test accuracy. The 95% CI of sensitivity and specificity was calculated using the exact method. The kappa statistic was calculated to evaluate the agreement between results from the TB-CX tests, conventional methods, and the molecular assay. A TB-CX test result was compared to the reference LJ culture method for MTB detection. The TB-CX test method was also tested using LJ indirect proportional DST and MGIT methods as gold standards for DST pattern of INH, RIF and PZA respectively. In addition, a GeneXpert Real-time PCR based molecular method was used to test all isolates. The turnaround time of the TB-CX test was compared to the gold standard test. In all analyses, p<0.05 was taken to indicate statistical significance.

## Results

### Patients and samples

A total of 147 human participants were enrolled in the study. Ten subjects were excluded: Five had contamination on their sputum LJ culture, three had insufficient *M*.*tb* growth on LJ culture, and two did not have all three tests results (TB-CX test, GeneXpert and LJ culture). Thus, a total of 137 study participants underwent final analysis: 88 (64.2%) were new TB cases and 49 (35.8%) were re-treatment cases. The median age of the participants was 30 years old (range 8–72) and 56% were male.

### *M*.*tb* growth detection

Among the 137 study participants, 78 patients were culture-positive by LJ and 59 patients were culture-negative by LJ (Table 1). Seventy-four patients were positive by TB-CX test. Fig 1 demonstrates growth of red *M*. *tb* colonies for drug susceptible TB. 87 patients were positive by GeneXpert. The TB-CX test detected 94% (73/78) of the LJ positive cultures, whereas the GeneXpert detected 96% (75/78) of the LJ positive cultures. Of the 59 LJ `negative cultures, the TB-CX test was negative in 58 (98%), while GeneXpert was negative in 47 (80%). The TB-CX test showed a strong agreement with both the LJ culture (96%, kappa 0.91) and GeneXpert (89%, kappa 0.78) for the detection of *M*.*tb*.

**Table 1:** Diagnostic accuracy of the tuberculosis culture (TB-CX) test, Xpert MTB/RIF test compared to the Lӧwenstein Jensen (LJ) culture method.

Assay				Sensitivity	Specificity	PPV	NPV	Kappa	Test Efficiency	P-value
	Solid LJ culture									
**TB-CX Test**	Positive	Negative	Total							
Positive	73	1	74							
Negative	5	58	64	94%	98%	99%	92%	0.91	0.96	< 0.001
Total	78	59	137							
**GeneXpert**										
Positive	75	12	87							
Negative	3	47	50	96%	80%	87%	98%	0.80	0.89	< 0.001
Total	78	59	137							

**Note**: PPV = positive predictive value, NPV = negative predictive value, LJ = Lӧwenstein-Jensen, the kappa **(k)** value is a measure of test reliability, interpreted as follows: <0.4 = poor; 0.4–0.75 = fair to good; >0.75 = strong.

### Drug-resistant TB detection

A total of Seventy eight LJ culture positive *M*.*tb* isolates were subjected to reference DST by LJ and eight were excluded: Three due to contamination, two due to failure to grow on reference DST, and three due to lack of TB-CX testing.

#### INH resistance detection

For detection of INH drug resistance, the TB-CX test and LJ DST were in agreement for 65 samples (93% concordant; 44 susceptible, 21 resistant; kappa = 0.84). Among the five discordant samples, two *M*.*tb* isolates were INH-susceptible by TB-CX test but resistant by LJ DST. Three isolates were INH resistant by TB-CX test and INH susceptible by LJ indirect proportional DST. The sensitivity of the TB-CX test for INH was 91% and specificity was 94% (Tables 2 and 3).

**Table 2:** Comparison of drug susceptibility test results for sputum samples from tuberculosis patients as determined by the tuberculosis culture (TB-CX) and Lӧwenstein Jensen (LJ) indirect proportional DST (n = 70).

TB-CX test	LJ DST		Agreement		Kappa Value	p value
	Susceptible	Resistant	Concordant	Discordant		
**INH**						
Susceptible	44 (94%)	2(9%)	65 (93%)	5 (7%)	0.84	< 0.001
Resistant	3(6%)	21(91%)				
**RIF**						
Susceptible	19 (40%)	0 (0%)	42 (60%)	28 (40%)	0.31	< 0.001
Resistant	28(60%)	23(100%)				
**MDR**						
Susceptible	48 (94%)	2 (10%)	65 (93%)	5 (7%)	0.82	< 0.001
Resistant	3(6%)	17 (90%)				
	**MGIT DST**					
**PZA**	Susceptible	Resistant				
Susceptible	17(85%)	0 (0%)	31 (91%)	3(9%)	0.82	< 0.001
Resistant	3(15%)	14(100%)				

**Note**: LJ IPM: LJ indirect proportional DST; MGIT: Mycobacterial Growth Indicator Tube; the kappa (k) value is a measure of test reliability, interpreted as follows: <0.4 = poor; 0.4–0.75 = fair to good; >0.75 = strong.

**Table 3:** Performance characteristics of the tuberculosis culture (TB-CX) test compared to the Lӧwenstein Jensen (LJ) indirect proportional drug susceptibility test (DST) for detection of drug-tesistant *M*. *tuberculosis* (*M*.*tb*) isolates (n = 70).

Test Drugs	N	Prevalence	Sensitivity	Specificity	PPV	NPV	Test Efficiency
INH	70	23 (33%)	91%	94%	88%	96%	0.93
RIF	70	23(33%)	100%	40%	45%	100%	0.60
MDR	70	19(27%)	90%	94%	85%	96%	0.93
PZA	34	14(41%)	85%	100%	100%	82%	0.91

**Note**: INH: isoniazid; RIF: rifampicin; PZA: Pyrazinamide; PPV: positive predictive value; NPV: negative predictive value; *M*.*tb*: *Mycobacterium tuberculosis*; MDR: Multidrug resistance to INH and RIF

#### RIF resistance detection

For RIF drug resistance testing, the GeneXpert test was in agreement with LJ DST for 69/72 *M*.*tb* isolates (96% concordant). However, of 70 isolates tested for RIF resistance, the TB-CX test was in low agreement with the GeneXpert test for detection of RIF drug resistance (57% concordant; kappa 0.28) (Table 4). For RIF drug resistance, the TB-CX test and LJ DST were in agreement for 42 samples (60% concordant; 19 susceptible, 23 resistant; kappa 0.31). The 28 discordant samples were all RIF resistant by the TB-CX test and RIF susceptible with LJ DST. The sensitivity of the TB-CX test for RIF was 100% and specificity was 40% (Tables 2 and 3).

**Table 4:** Comparison the LJ indirect proportional method (n = 72), the tuberculosis culture (TB-CX) test (n = 70) with Xpert MTB/RIF assay for detection of rifampin drug resistance.

	XpertMTB/RIF		Agreement		kappa value	P value
	Susceptible	Resistance	Concordant	Discordant		
**LJ IPM (RIF)**						
Susceptible	49 (98%)	2 (9%)	69(96%)	3(4%)	0.87	< 0.001
Resistance	1 (2%)	20 (91%)				
**TB-CX test**						
Susceptible	6 (12%)	0 (0%)	27(39%)	43(61%)	0.28	< 0.001
Resistance	43 (88%)	21(100%)				

**Note**: LJ IPM: LJ indirect proportional DST, the kappa **(k)** value is a measure of test reliability, interpreted as follows: <0.4 = poor; 0.4–0.75 = fair to good; >0.75 = strong.

#### MDR resistance detection

**Fig 2:**
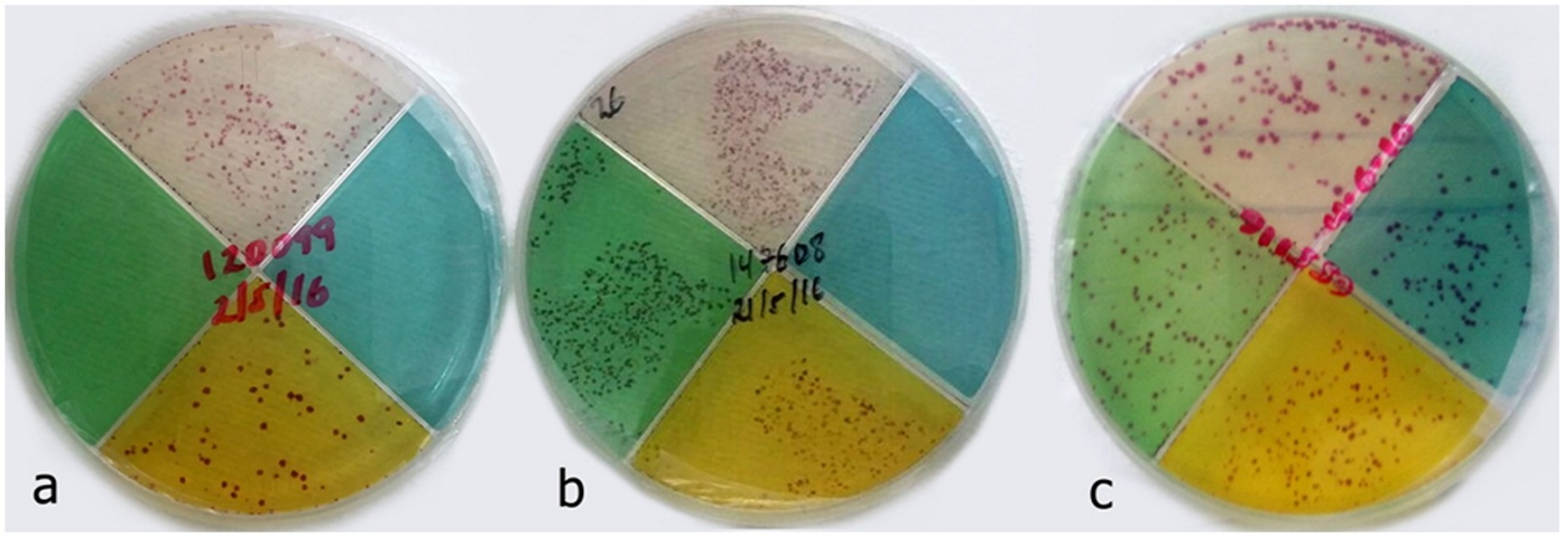
Drug-resistant *Mycobacterium tuberculosis* (*M*.*tb*) detection using the TB-CX test. RIF resistant, *M*.*tb* red colony growth on clear and yellow quadrants (a); MDR-*M*.*tb*, *M*.*tb* red colony growth on clear, green, and yellow quadrants (b); MDR-M.tb + PZA resistant, *M*.*tb* red colony growth on clear, green, yellow, and blue quadrants (c).

A total of 70 specimens were tested for INH and RIF drug susceptibility. Of the 73 isolates tested using the TB-CX test, 20 (15%) had MDR-TB (resistance to both INH and RIF). The TB-CX test detected combined resistance to INH, RIF, and PZA (MDR-TB plus PZA) in 13 isolates, all of them belonging to previously treated patients (Fig 2). For MDR-TB detection, the TB-CX test and LJ DST were in agreement for 65 samples (93% concordant; 48 susceptible, 17 resistant; kappa 0.82) (Table 2). The sensitivity of the TB-CX test for MDR-TB was 90% and specificity 94% (Table 3).

#### PZA resistance detection

The sensitivity and specificity of the TB-CX test to detect drug resistance to PZA compared to the MGIT DST were 85% and 100%, respectively. The agreement between the TB-CX test and MGIT DST for detection of PZA resistant TB was 91% (kappa 0.82). Of the 38 samples tested for PZA DST, 34 (90%) of the TB-CX tests were interpretable and thus compared to the MGIT DST. There was agreement between PZA DST by TB-CX and MGIT for 31/34 samples (91% concordant; 17 susceptible, 14 resistant, kappa = 0.82). The three discordant PZA samples were PZA resistant with the TB-CX test but susceptible using the MGIT DST. TB-CX test sensitivity and specificity to detect PZA were 85% and 100%, respectively (Tables 2 and 3).

### Turnaround time (TAT) for the TB-CX test

The time elapsed from the date of inoculation to the date of concurrent growth detection and susceptibility results for each sample was registered as the TAT for the TB-CX test (Table 5). The median TAT results by TB-CX test was 13 days (range 5–23 days). In contrast, the reference LJ-culture based indirect DST required a median of 50 days for DST, 28 days (range 12–46 days) for culture growth and then an additional 22 days (range 15–35) to determine drug susceptibility using LJ indirect proportional method.

**Table 5:** Frequency table of tuberculosis culture (TB-CX) Test Turnaround Time (TAT).

TB-CX) Test TAT (no. of days)	Frequency (n)	%	Cumulative %
5	2	2.7	2.7
9	6	8.2	10.9
11	12	16.4	27.3
13	18	24.7	52.0
15	24	32.9	84.9
17	6	8.2	93.1
19	3	4.1	97.2
21	1	1.4	98.6
23	1	1.4	100.00
Total	73	100.00	

## Discussion

Rapid determination of *M*.*tb* drug susceptibility is important for patient management and public health. Unfortunately, the utility of current TB diagnostic modalities in low and middle-income countries is limited either by turnaround time (traditional solid media) or by affordability, infrastructure, and/or technical expertise (liquid media systems and molecular diagnostics). Our present study revealed that an inexpensive TB-CX test had rapid TAT and comparable sensitivity for the detection of *M*.*tb* as well as isoniazid, rifampin, and pyrazinamide susceptibility when compared to LJ culture/indirect proportional method/MIGT and the GeneXpert test.

The TB-CX test had a sensitivity of 94% and specificity of 98% for growth and detection of *M*.*tb* when compared to solid media LJ culture. Furthermore, the median time to detection of *M*.*tb* isolates with determination of susceptibility was 13 days for TB-CX, compared to a median turnaround time to growth of 28 days for LJ culture, with an additional 22 days for DST results (total median of 50 days). The median TAT for TB-CX in this study was comparable to previously reported values of 10 to 13 days [5, 12, 13, 22]. Interestingly, the fastest growth was observed with two sputum samples on day 5 of incubation and results for 85% of 73 TB-CX culture-positive sputa were available within 15 days. The color contrast provided by the red *M*.*tb* colonies makes them observable to the naked eye at early stages of growth or micro-colonies in 2 weeks instead of the normal 3 weeks incubation [24].

Molecular assays such as the GeneXpert are rapid, simultaneously detecting *M*.*tb* and RIF drug resistance within two hours [18, 25]. Our findings indicate that GeneXpert assay has a strong agreement with solid LJ culture for detection of *M*.*tb*. GeneXpert detected 96% (75/78) of the LJ positive cultures. The three LJ positive isolates were further confirmed by colony morphology and positive acid-fast bacilli using Ziehl-Neelsen (ZN) staining method. Of these three LJ positive isolates, two of them were also positive by the TB-CX test. However, the specificity of GeneXpert assay for the detection of *M*.*tb* was 80% when compared to LJ culture. The specificity discrepancy between GeneXpert and LJ culture might be attributed to anti-TB medication prior to obtaining specimen collection. Among the 9 GeneXpert positive patients with LJ culture negative, seven of them were on anti-TB drugs (range of 3 to 7 days into treatment). Anti-TB drugs kill and reduce the live TB bacilli load per ml of sputum, and although the genes of dead MTB bacilli might be detected by GeneXpert, these are not detected by LJ, which requires 10–100 viable bacilli per ml of sputum [8, 18]. Moreover, it is also possible a variation in *M*.*tb* bacilli load between the sputum used for GeneXpert and the LJ cultures, though paired morning expectorated sputum samples were collected on the same day from the same patient [26, 27].

Even though the price of the GeneXpert assay has been reduced in many low-and middle-income countries, cost remains prohibitive in some resource-constrained settings. Relatively the cost of TB-CX test per test is minimal (4–5 US$). Another advantage of the TB-CX test in comparison to GeneXpert assay is the ability to detect two additional drugs in addition to RIF, such as INH and PZA. Although both testing modalities require a stable power source, relative disadvantages of the TB-CX include access to and maintenance of a refrigerator and an incubator.

Reported INH resistance for Ethiopia was 3.2% in new cases and up to 56% in retreatment cases [28]. For patients with INH mono-drug resistance, the continuation phase of drug treatment would be extended to 9 months and would include the combination of RIF, PZA, and EMB rather than RIF and INH alone. Thus, if INH drug resistance is not known, RIF drug resistance and the development of MDR-TB can occur In this study, the DST agreement between TB-CX test and the reference susceptibility method was excellent (kappa value of 0.84 and concordance above 90%) in detection of INH resistance. However, we observed a relatively lower INH resistance detection rate compared to previous assessments using TB-CX test and TLA tests, which yielded 98% to 100% sensitivity and specificity for detection of INH resistance [12, 29]. As opposed to indirect inoculates (isolates), direct inoculation of sputum is associated with lower sensitivity and specificity and may have contributed to our results compared to published data [5, 29].

Despite being simple and rapid to use, the new color plate test has an important limitation, namely poor specificity for RIF resistant isolate detection. The TB-CX test misclassified nearly half of the RIF susceptible isolate as RIF resistant. Other studies have reported 98% sensitivity and 88% specificity for RIF resistance detection with 96% agreement using TB-CX test [12] and 100% sensitivity and specificity for RIF resistance detection recorded using TLA assay on both direct and indirect specimens [5, 29, 30]. The difference may be due to variation in specimen types. We prospectively enrolled subjects and processed sputum collection directly for decontamination and inoculation onto TB-CX plate. Transport and field conditions in Ethiopia may have degraded RIF drug in the quadrant, resulting in aberrant increased growth. Logistical constraints in Ethiopia caused the TB-CX Test to be transported at ambient temperature and to be stored for up to 4 months, contrary to the refrigeration and 66-day shelf-life required by the protocol which may have degraded RIF drug in the quadrant, potentially explaining the aberrant growth in the rifampicin containing quadrant that we observed in 40% of cultures.

The concentration of RIF was 1μl/ml and the batch of TB-CX plates were validated in the laboratory in the US prior to shipment. Other authors have noted rare discordant results from phenotypic drug susceptibility testing compared to genotypic testing when GeneXpert or other molecular tests were compared to culture due to silent rpoB mutation, missense mutation, or low-level resistance to RIF [31–34]. Due to the low specificity of the TB-CX (40% only) to detect RIF resistance *vs*. the other tests evaluated, further studies need to be performed to clarify if the TB-CX test can effectively be used for RIF resistance detection, including building on-site capabilities in Ethiopia to make the TB-CX test to bypass potential RIF on plate degradation issues during transport to the testing sites and/or in-site prolonged storage at suboptimal temperature conditions.

A disadvantage of the TB-CX plate as far as staff requirements is that the rapid time to growth requires reading of the plate for culture detection three times a week, opposed to once a week for LJ. However, the less-frequent interval of once per week for LJ culture tube reading may result in limited detection of *M*.*tb* growth in-between reads. This weekly interval was chosen based on current Ethiopia National TB and WHO TB guidelines.

PZA in combination with INH, RIF, and EMB is the WHO-recommended first-line drug therapy for drug-susceptible TB. The addition of PZA to INH and RIF in the initiation phase allows for shortening the previous duration of therapy of 9–12 months to the current 6 months for drug- susceptible TB [35, 36]. If a patient is not able to take PZA in the first two months, either due to intolerance or PZA drug resistance, then the treatment course is extended from 6 months to 9 months due to increased treatment relapse. So, the ability to detect PZA resistance may be of benefit to improving outcomes of patients with drug susceptible TB. In the current study, the TB-CX test detected PZA drug resistance with 85% sensitivity and 100% specificity 100% and had strong agreement rates (kappa value, 0.82; concordance, 91%) compared to MGIT.

An additional advantage of the TB-CX test is low biohazard risk in the laboratory. The TB-CX test plates were double-sealed after initial sputum inoculation. Reading and disposal of the culture plate performed without breaking of the Parafilm seal or removal from the Zip-lock bag, decreasing handling risks. In addition, because of the shorter TAT for TB-CX results, the potentially bio-hazardous TB-CX materials are discarded in less time than in conventional methods.

Lastly, the TB-CX test also has an optimal operational characteristic. Due to the color contrast nature of the TB-CX test, it is possible to carry out daily faster checking of the test visually or with light microscopy for micro-colony detection. Another innovation of the TB-CX test is that the drug containing quadrants are flexible enough to be adjusted to provide rapid MDR-/pre-XDR-TB diagnosis with three drug containing quadrants (INH, RIF, fluoroquinolone) with one format or diagnosis of bovine TB (i.e. switching fluoroquinolone for PZA). Previous TB-CX test using ciprofloxacin (CIP) found excellent accuracy of CIP resistance detection, which is a marker for pre-XDR-TB [5, 12]. This is the first published TB-CX test with detection of PZA resistance. PZA drug resistance is a surrogate marker for bovine TB and can be utilized in areas with increased concern for zoonotic TB transmission.

Despite providing rapid diagnosis, comparatively low cost per test result, and apparent simplicity of use, the TB-CX test has limitations. One of the technical limitations could be lack of specificity of micro-colony cording detection, unable to differentiate MTB from non-tuberculosis mycobacterium (NTM) particularly *M*. *chelonae* that also exhibits cording [5]. However, along with the characteristic micro-colony morphology of MTB observed, the inclusion of Paranitrobenzoic Acid (PNB)-containing quadrants in the TB-CX test, which specifically inhibit NTM growth, was added as a solution to ruling out false-positive results produced by NTM growth [5, 37].

## Limitation of the study

This study presented several limitations. An internal quality control was done against physical appearance and sterility of all the TB-CX plates. Despite the fact that concentrations (stability) of drugs in TB-CX plates were not concurrently validated (culturing the plate while quality control was underway), few TB-CX plates (3%) were tested with control strains just after transit in the field. Since the plates had been stored up to 4 months prior to inoculation, degradation of the drugs on TB-CX plates with long term suboptimal temperature storage conditions and during transport (overseas and in-country transport to testing sites) could be possible. Sputum specimens were not cultured right away but frozen (-20°C) for 14 days which may also decrease the sensitivity of the TB-CX test. Unlike conventional culture method, a diluted sputum-disinfectant mixture was used as the inoculum. All of these factors may lead to decrease the sensitivity of TB-CX test. The other drawback of the study was the lack of comparative PZA DST by MGIT for all LJ culture positive isolates due to resource limitations.

## Conclusion

In this study, we evaluated a rapid DST method that concurrently detects *M*.*tb* and susceptibilities to INH, RIF and PZA, the three key first-line anti-TB drugs used in the treatment of TB. Despite the suboptimal performance for detection of RIF resistance (40%), the TB-CX test showed similar sensitivity for detecting *M*.*tb* and selective drug resistant-TB in a timely way as compared to solid media and GeneXpert. Limitations of this test need to be further evaluated to increase the power of the analysis by increasing the number of samples tested before further implementation in clinical settings.
